# Trends and factors associated with adolescent pregnancies in Tanzania from 2004-2016: Evidence from Tanzania Demographic and Health Surveys

**DOI:** 10.24248/eahrj.v7i1.707

**Published:** 2023-07-12

**Authors:** Octavian Aron Ngoda, Jenny Renju, Michael Johnson Mahande, Sophia Adam Kagoye, Innocent Baltazar Mboya, Sia Emmanueli Msuya

**Affiliations:** a Department of Epidemiology and Biostatistics, Institute of Public Health, Kilimanjaro Christian Medical University College, Moshi Tanzania; b Tanzania Medicine and Medical Devices Authority, Dodoma, Tanzania; c Department of Population Health, London School of Hygiene and Tropical Medicine, London, UK; d School of Mathematics, Statistics & Computer Science, University of Kwazulu-Natal, Pietermaritzburg, South Africa; e Department of Community Health, Institute of Public Health, Kilimanjaro Christian Medical University College, Moshi, Tanzania; f Department of Community Medicine, Kilimanjaro Christian Medical College Hospital, Moshi, Tanzania.

## Abstract

**Background:**

Adolescent pregnancy increases the risk of maternal and child morbidity and mortality. We aimed to determine trends and factors associated with adolescent pregnancy in Tanzania from 2004 to 2016 using the Tanzania Demographic and Health surveys (TDHS).

**Methods:**

We carried out an analytical cross-sectional study using the TDHS data for the years 2004 to 2005, 2010 and 2015 to 2016 among adolescent girls aged 15 to 19 years. Data analysis was performed using STATA version 15. Data analysis considered the complex survey design inherent in the demographic and health survey (DHS) data. The Poisson regression model was used to estimate Prevalence Ratios (PR) and 95% confidence intervals for factors associated with adolescent pregnancy.

**Results:**

We analysed data for a total of 10,972 adolescents for the three TDHS rounds. The proportion of adolescent pregnancy significantly decreased from 26% to 22.8% from the year 2004/05 to 2010 and then increased again to 26.7% in 2015/16. Adolescents who were aged 18 to 19 years (APR 1.52; 95% CI, 1.38 to 1.68) married or cohabiting with their partners (APR 2.15; 95% CI, 1.93 to 2.40; *P<.001*), widowed/divorced/separated (APR 2.32; 95% CI, 2.03 to 2.66; *P<.001*), and among those who started sexual activity before 15 years of age (APR 1.20; 95% CI, 1.11 to 1.31; *P<.001*) were more likely to become pregnant during adolescence. In contrast, adolescents with secondary school education level and above were the least likely to become pregnant (APR 0.62; 95% CI, 0.51 to 0.75; *P<.001*) compared to those with no formal education.

**Conclusion:**

One in four adolescent girls aged 15 to 19 in Tanzania have already started childbearing and despite fluctuation, high rate of adolescent pregnancy persists. Preventive interventions should focus on adolescents with low education level, married/cohabiting with their partners, and who have started sex before 15 years of age. We advocate for the increase of school attendance until high school level to reduce the risk of early pregnancy in adolescents. Furthermore, qualitative studies are crucial to explore reasons for the rising trend of adolescent pregnancy in most zones of Tanzania, particularly between 2010 and 2015/16.

## BACKGROUND

Adolescent pregnancy is a pregnancy occurring in girls aged 10 to 19 years.^1,2^ Globally, one in five girls give birth by the age of 18, which contributes to about 11% of total births worldwide,^2^ with approximately 95% of adolescent births occurring in low- and middle-income countries, and over 50% of women give birth before the age of 20 years in sub-Saharan Africa (SSA).^3^

Tanzania is among the countries in SSA with the highest rate of adolescent pregnancy, which has been increasing over time from 23% in 2010 to 27% in 2016).^6^ Factors that influence adolescent pregnancies vary with settings. For example, in Nepal, poverty, unemployment and low education levels of both the adolescents and their partners were identified as key drivers of adolescent pregnancy.^7^ A systematic review in Africa revealed that rural residence, ever married, lack of parent to adolescent communication on sexual and reproductive health (SRH) issues and no women education was associated with adolescent pregnancy.^8^ Other factors such as coercive sexual relations, gender power relations, poverty early marriages, use of alcohol, substance abuse, cost of contraceptives and misconceptions about contraceptives and non-friendly adolescent reproductive services were reported in other systematic reviews in a Sub Saharan Africa (SSA).^9,10^

 Various interventions have been implemented to reduce adolescent pregnancy in Tanzania, such as strategies to offer Adolescent-Friendly Sexual Reproductive Health Services (AFSRHS) which provide free contraceptive services, family planning education, and comprehensive sexual education.^11,12^ Coverage of AFSRH services is reported to have increased from 30% in 2010 to 63% in 2017, but far from the national target of 80% by 2020.^12,13^ In 2019 the country started 5 years (2019 to 2022) National Accelerated Investment Agenda for Adolescent Health & Wellbeing (NAIA), which is implemented by several ministries.^14^ NAIA has six pillars, of which the second is preventing teenage pregnancies.^14^ Despite these interventions, the country is far from reaching the national target of reducing the adolescent fertility rate to less than 90 births per 1000 adolescents by 2020.^12^

In Tanzania, studies on factors associated with adolescent pregnancies using national-level data are limited. Previous studies that investigated factors associated with adolescent pregnancy were at the subnational level. Tanzania and Demographic Health Survey (TDHS) reports also give information on the prevalence of adolescent pregnancies but do not analyze predictors of adolescent pregnancies. Information on change of factors associated with adolescent pregnancies over time is therefore lacking. This analysis aimed to determine trends and factors associated with adolescent pregnancies in Tanzania from 2004 to 2016 using the TDHS data. Results from this study will assist the policymakers and program managers to frame an integrated strategy and programmatic response to reduce adolescent pregnancies and improving their health and socio-economic life.

## METHODS

### Study design and setting

This was an analytical cross-sectional study conducted using nationally representative secondary data from the three TDHS of 2004/2005, 2010 and 2015/2016 respectively. The study included data from all 31 regions of Tanzania. Tanzania is one of the largest countries in Africa, covering 947,300 square kilometers. According to the 2012 census, the population was 44.9 million, of which 9.9 million (23%) were adolescents between 10 and 19 years of age. The average annual growth rate according to the 2012 population and housing census was 2.7%.^15^ The average fertility rate in Tanzania was estimated to be 5.2 children born per woman, and mothers' mean age at first birth was 19.8 years.^6^

### Study Population, Sample Size, and Sampling

The TDHS used a multistage sampling technique in selecting study participants, which was designed to provide estimates for the entire country, for urban and rural areas on the mainland and Zanzibar. This sampling design was guided by the considerations of the availability of an existing sampling frame to get the full coverage of the target population.

Stage one involved the selection of a stratified sample from a list of enumeration areas (EAs) that had been obtained from the recent census conducted in Tanzania. These EAs were the clusters. In rural areas, an EA is a natural village, or a segment of a large village, or a group of small villages; in urban areas, an EA is a street or a city block. This sample of EAs was selected with considerations of probability proportional to size (PPS) that takes into account the size of the enumeration area. A listing procedure was then performed on each of the selected EAs such that all dwellings and households are listed.

In the second stage, a complete list of households available in each of the selected EAs, a fixed variable number of households, was selected by equal probability systematic sampling technique. In each of the selected households, a questionnaire was then completed to identify women aged 15 to 49 years. Every eligible woman was then interviewed.

**FIGURE 1:**
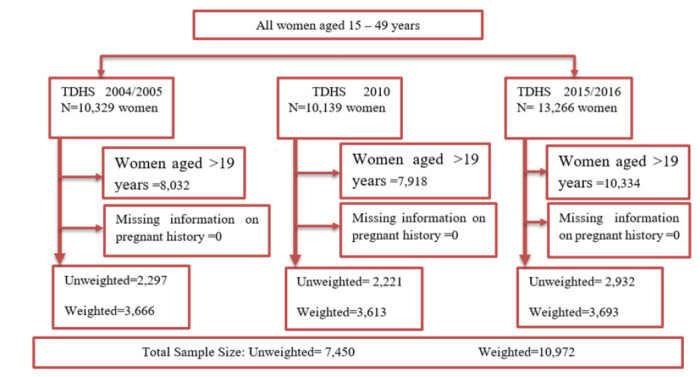
Flow Chart for the Selection of Study Participants “For Adolescent Pregnancies”

In this study, the population included in the analysis consisted of adolescent girls aged 15 to 19 years. Adolescents with missed records on birth history were excluded from the study since they did not have their pregnancy records. This resulted in a total weighted sample of 10,964 from the three TDHS; 2,297 adolescents in 2004/2005 (3,666 weighted cases), 2,221 adolescents in 2010 (3,613 weighted cases) and 2932 adolescents in 2015/2016 (3,693 weighted cases) (Figure 1).

### Study Variables and Variable Measurements

In this study, the dependent variable was an adolescent pregnancy. This was a binary variable coded “Yes” if an adolescent girl reported to have ever had a birth or was pregnant at the time of the interview and “No” if she reported the contrary. This definition was adapted from TDHS as this information is consistently available in all the TDHS. The independent variables in this study included socio-demographic and sexual and reproductive health characteristics. Socio-demographic characteristics include adolescent age in years (15 to 17, 18 to 19), working status (working, not working), education level (no education, primary, secondary and above), wealth index categories (poorest, poorer, middle, richer, richest), marital status (never in a union, married/cohabiting, widowed/divorced/separated), residence (urban-rural), geographical zones (western, northern, central, southern highlands, southern, south-west highlands, lake, eastern, Zanzibar), and partner education level (no education, primary, secondary and above). Reproductive health characteristics include age at first sex in years (<15, ≥15), age at first marriage in years (<15, 15 to17, 18 to 19) and modern contraceptive use (No, or Yes).

### Statistical Analysis

Data analysis, using STATA version 15, accounted for the complex nature of survey design through the application of weights, primary sampling unit (cluster) and strata for the adjustment of the cluster sampling survey design. Descriptive statistics were summarized using frequency and proportions for categorical variables and continuous variables using mean and median, and respectively standard deviation and interquartile range (IQR).

Poisson regression analysis was used to determine factors associated with adolescent pregnancy as an alternative to the classical logistic regression, as the proportion of adolescent pregnancies was greater than 10%. The choice of this model was also motivated by the non-convergence of the log-binomial regression model.

The Poisson regression model estimated prevalence ratios (PR) with their 95% CI for factors associated with adolescent pregnancy. Bivariate Poisson regression was conducted to examine the unadjusted association between exposure variables and the likelihood of adolescent pregnancies. Variables with a *p-value* <.05 in the bivariate analysis were entered in the multivariable model to adjust for the potential confounding effect. We used stepwise forward elimination regression methods for model building. Models with the lowest Akaike information criteria (AIC) were regarded as more parsimonious, hence more suitable to explain how independent and dependent variables are associated. Multicollinearity was evaluated among the explanatory variables to be included in the Poisson regression model by inspecting the correlation matrix and assessing estimation problems of the model parameters.

### Ethical Considerations

Ethical approval was obtained from the Kilimanjaro Christian Medical University College Research and Ethics Review Committee (CRERC no.PG/012/2019). The parent study obtained written, informed consent from the study participants. Interviews were conducted in a private place around the household, and participants were identified using unique identification numbers to ensure confidentiality and privacy of participant information. Permission to use the TDHS data was obtained from the Demographic Health Survey program. Data was used solely for the current study.

## RESULTS

### Background Characteristics of Study Participants

Background characteristics of the study participants are shown in Table 1. Data were analyzed for a total of 10,972 adolescents in this study. The proportion of adolescents who were working decreased across the survey years from 57.6% in 2004/2005, to 49.1% in 2010 and 45.0% in 2015/2016. The proportion of participants having a secondary or above education level increased from 11.4% in 2004/2005 to 34.5% in 2010 and 35.1% in 2015/2016. The proportion of adolescents who resided in rural areas remained constant between 2004/2005 (70.2%) and 2010 (70.4%) but then decreased to 62.7% in 2015/2016.

**TABLE 1:** Background Characteristics (weighted) of Study Participants in Tanzania Demographic and Health Survey 2004/2005, 2010 and 2015/2016 (N=10,972) in Different Zones

Variable	TDHS Survey Year		
	2004/05 (n=3666) n (%)	2010 (n=3613) n (%)	2015/16 (n=3693) n (%)
Women Age (Years)			
15–17	2,206 (60.2)	2,322 (64.3)	2,165 (58.6)
18–19	1,460 (39.8)	1,291 (35.7)	1,528 (41.4)
Mean ± SD	17.0 ± 1.4	17.0 ± 1.4	17.0 ± 1.5
Working status			
Not working	1,552 (42.4)	1,820 (50.9)	2,027 (55.0)
Working	2,110 (57.6)	1,759 (49.1)	1,657 (45.0)
Education level			
No education	757 (20.6)	300 (8.3)	2,22 (6.0)
Primary	2,491 (68.0)	2,068 (57.2)	2,175 (58.9)
Secondary and above	418 (11.4)	1,245 (34.5)	1,296 (35.1)
Wealth index			
Poorest	580 (15.8)	442 (12.3)	640 (17.3)
Poorer	733 (20.0)	655 (18.1)	587 (15.9)
Middle	673 (18.4)	718 (19.9)	596 (16.2)
Richer	639 (17.4)	817 (22.6)	774 (21.0)
Richest	1,041 (28.4)	979 (27.1)	1,096 (29.6)
Marital status			
Never in union	2,642 (72.1)	2,902 (80.3)	2,759 (74.7)
Married/living together	963 (26.3)	664 (18.4)	849 (23.0)
Widowed/divorced/separated	61 (1.6)	47 (1.3)	85 (2.3)
Place of residence			
Urban	1,095 (29.8)	1,070 (29.6)	1,377 (37.3)
Rural	2,572 (70.2)	2,543 (70.4)	2,316 (62.7)
Geographical zones			
Western	445 (12.1)	319 (8.8)	412 (11.1)
Northern	428 (11.7)	456 (12.6)	426 (11.5)
Central	370 (10.1)	344 (9.5)	332 (9.0)
Southern highlands	234 (6.4)	242 (6.7)	186 (5.1)
Southern	178 (4.9)	167 (4.6)	150 (4.1)
Southwest	360 (9.8)	308 (8.5)	341 (9.3)
Lake zone	993 (27.1)	1109 (30.7)	1,080 (29.2)
Eastern	533 (14.5)	536 (14.8)	642 (17.4)
Zanzibar	125 (3.4)	132 (3.8)	124 (3.3)
Partner education level			
No education	241 (23.5)	142 (20.0)	117 (13.8)
Primary	719 (70.2)	512 (72.3)	564 (66.5)
Secondary and above	64 (6.3)	54 (7.7)	167 (19.7)
Age at first sex (years)			
<15	351 (23.1)	345 (26.6)	472 (24.5)
≥15	1,172 (76.9)	950 (73.4)	1,454 (75.5)
Median (IQR)	15 (2)	15 (2)	15 (3)
Modern contraceptive use			
No	1,312 (87.9)	941 (74.7)	1,544 (83.0)
Yes	180 (12.1)	318 (25.3)	316 (17.0)

a Frequencies do not tally with the total due to missing values in these variables

Abbreviation: TDHS, Tanzania Demographic and Health Survey

Nearly one in four adolescents (23.1% to 26.6%) in the three surveys reported starting sex at age <15 years. The proportion of adolescents who were married/cohabited decreased from 26.3% in 2004/2005 and 18.4% in 2010 but then increased to 23.0% in 2015/2016. The proportion of adolescents who were using contraceptives increased from 12.1% in 2004/2005 to 25.3% in 2010 and then decreased to 17.0% in 2015/2016.

### The Trend of Adolescent Pregnancy Between 2004/2005 to 2015/16

**FIGURE 2:**
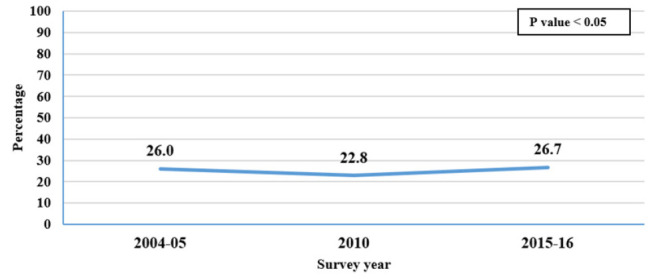
Trends of Adolescent Pregnancies from DHS 2004/05 to 2015/2016

The proportion of adolescent pregnancy significantly decreased from 26% to 22.8% between 2004/05 and 2010, and then increased again to 26.7% in 2015/16 (Figure 2).

**FIGURE 3:**
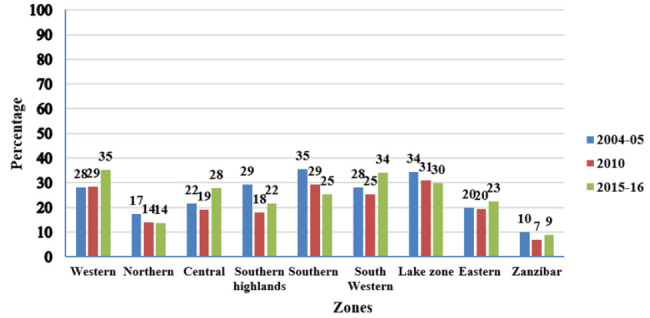
Prevalence of Adolescent Pregnancies by Zones from DHS 2004/05 to 2015/2016

Between 2004/05 and 2010 TDHS rounds, adolescent pregnancy decreased in seven zones, namely northern, central, southern highland, southern, south-western, lake, and Zanzibar. Contrastingly, between 2010 and 2015/16 adolescent pregnancy decreased in only two zones: southern, and lake zones, while it remained the same in the northern zone, (Figure 3).

### Factors Associated with Adolescent Pregnancy Between 2004/05 – 2015/16

In the bivariate Poisson regression analysis, adolescent age, employment status, education level, household wealth index, marital status, geographical zones, place of residence, contraceptive use, age at first marriage, and age at first sexual intercourse were significantly associated with adolescent pregnancy. In multivariable analysis, adolescent age, education level, marital status, and age at first sexual intercourse were the independent predictors of adolescent pregnancy after adjusting for other variables, (Table 2).

**TABLE 2:** Factors Associated With Adolescent Pregnancy in Tanzania, Tanzania Demographic and Health Survey 2004/2005, 2010 and 2015/2016 (N=10972)

Variable	Total	Pregnant n (%)	CPR (95% CI)	P Value	APR (95% CI)	P Value
Women age (years)						
15–17	6,694	831 (12.4)	1		1	
18–19	4,278	1,932 (45.2)	3.64 (3.25, 4.07)	<.001	1.52 (1.38, 1.68)	<.001
Working status						
Not working	5,400	771 (14.3)	1		1	
Working	5,527	1,982 (35.9)	2.51 (2.24, 2.81)	<.001	1.06 (0.97, 1.17)	.204
Education level						
No education	1,278	596 (46.6)	1		1	
Primary	6,735	1,941 (28.8)	0.62 (0.55, 0.69)	<.001	0.97 (0.88, 1.07)	.491
≥Secondary	2,959	226 (7.6)	0.16 (0.13, 0.20)	<.001	0.62 (0.51, 0.75)	<.001
Wealth index						
Poorest	1,662	581 (35.0)	1		1	
Poorer	1,974	661 (33.5)	0.96 (0.84, 1.09)	.517	1.05 (0.94, 1.16)	.389
Middle	1,988	558 (28.1)	0.80 (0.69, 0.94)	.006	1.08 (0.96, 1.21)	.187
Richer	2,230	533 (23.9)	0.68 (0.59, 0.80)	<.001	1.06 (0.94, 1.20)	.362
Richest	3,118	430 (13.7)	0.39 (0.33, 0.48)	<.001	0.87 (0.73, 1.04)	.122
Marital status						
Never in union	8,303	749 (9.0)	1		1	
Married/cohabiting	2,476	1,857 (75.0)	8.31 (7.41, 9.33)	<.001	2.15 (1.93, 2.40)	<.001
Widowed/divorced/separated	193	157 (81.9)	9.07 (7.84, 10.50)	<.001	2.32 (2.03, 2.66)	<.001
Place of residence						
Urban	3,542	625 (17.7)	1		1	
Rural	7,430	2,139 (29.0)	1.63 (1.41, 1.89)	<.001	0.95 (0.84, 1.08)	.458
Geographical zones						
Western	1,176	362 (30.8)	1.94 (1.52, 2.48)	<.001	0.99 (0.83, 1.19)	.929
Northern	1,310	208 (15.9)	1	1		
Central	1,046	263 (25.2)	1.59 (1.23, 2.03)	<.001	1.03 (0.85, 1.25)	.732
Southern highlands	662	149 (22.5)	1.42 (1.07, 1.89)	.016	1.16 (0.95, 1.43)	.152
Southern	495	149 (30.0)	1.89 (1.42, 2.52)	<.001	1.00 (0.82, 1.22)	.998
South west	1,009	293 (29.0)	1.83 (1.38, 2.42)	<.001	1.18 (0.97, 1.44)	.106
Lake zone	3,183	987 (31.0)	1.95 (1.58, 2.42)	<.001	1.00 (0.85, 1.18)	.999
Eastern	1,711	322 (18.8)	1.19 (0.90, 1.57)	.233	0.96 (0.78, 1.17)	.667
Zanzibar	380	30 (7.8)	0.49 (0.38, 0.64)	<.001	1.11 (0.90, 1.36)	.333
Modern contraceptive use						
No	3,797	1,910 (53.0)	1		1	
Yes	814	351 (43.1)	0.86 (0.75, 0.98)	.021	0.94 (0.85, 1.04)	.239
Age at first sex						
<15	1,168	662 (53.3)	1.13 (1.03, 1.24)	.009	1.20 (1.11, 1.31)	<.001
≥15	3,577	1,684 (47.1)	1		1	
Age at first marriage						
<15	395	328 (83.0)	1			
15-17	1,789	1,370 (76.6)	0.92 (0.86, 0.99)	.060		
18-19	485	317 (65.4)	0.79 (0.70, 0.89)	<.001		

Abbreviations: CPR, Crude Prevalence ratio; APR, Adjusted Prevalence Ratio

The prevalence of adolescent pregnancy was 52% higher among adolescents aged 18 to 19 years compared to those aged 15 to 17 years (APR 1.52; 95% CI, 1.38 to 1.68; *P<.001*). Adolescents with secondary education and above had a 38% lower prevalence of pregnancy compared to those with no formal education (APR 0.62; 95% CI, 0.51 to 0.75). Compared to adolescents who had never been in a union, those who were married or cohabiting and widowed/divorced/separated had 2.2 (APR 2.15; 95% CI, 1.93 to 2.40) and 2.3 (APR 2.32; 95% CI, 2.03 to 2.66) times higher prevalence of pregnancy. Adolescents who started sexual activity at age <15 years, had a 20% higher prevalence of pregnancy than those starting at age ≥15 years (APR 1.20; 95% CI, 1.11 to 1.31), (Table 2).

## DISCUSSION

The study aimed to determine the trend and factors associated with adolescent pregnancy in Tanzania from 2004 to 2016. We found that the prevalence of adolescent pregnancy has fluctuated in a 10 year period. Prevalence decreased from 26% in 2004/2005 to 22.8% in 2010 and increased again to 26.7% in 2015/2016, showing variation by geographical zones. Education level, adolescent age, marital status, and age at first sexual intercourse were independent predictors of adolescent pregnancies.

This study reported the fluctuating trend of adolescent pregnancies, which is consistent with a previous study in 5 East African countries (Kenya, Malawi Tanzania, Uganda, and Zambia),^16^ but different with findings in Nepal and Ethiopia which reported a decrease in the proportion of adolescent pregnancy over time.^8,17^ An upward trend in adolescent pregnancy during the latest survey could be caused by several policy level conversations and debates in the last few years. as it was observed that condom and contraceptive use among adolescents declined between 2010 and 2015/16 surveys. The proportion of adolescent girls using a condom at last sex declined from 50% to 37% between 2010 and 2015/16 surveys. The proportion of adolescents who were using contraceptives increased from 12.1% in 2004 to 25.3% and then decreased significantly to 17% in the 2015/16 survey. While the median age of debut remained the same at 15 years, the proportions of adolescent girls who were in the union and/or divorced or separated declined from 27.9% to 19.7% in 2010, then increased to 25.3 in 2015/16 respectively. Efforts to improve the availability and access to quality adolescent-friendly sexual and reproductive health services (AFSRHS) cannot be overemphasized in our setting. Coverage of AFSRHS has increased from 30% in 2010 to 63% in 2017, below the recommendation of 80%.^12,13^ Furthermore, the availability of quality and comprehensive adolescent and youth-friendly SRH services given by different stakeholders, and at the community level is needed.

In this study, adolescent girls with secondary education and above had a lower prevalence of adolescent pregnancies compared to those with no education. These findings are consistent with findings from Ethiopia and Nepal.^7,8,17^ Educated adolescents may be more empowered and better informed about their fundamental and legal rights that are essential to make a logical decision about healthy life, such as rejecting early marriage and early sexual intercourse. In the current study, only 4% of adolescents with secondary education were either married or cohabited with their partners. The findings are in contrast with the study done in Bangladesh that reported no significant protective effect of education against adolescent pregnancy.^18^ The small sample size (389), convenience sampling technique and the fact that older women aged >19 years constituted almost 74% of the study sample in Bangladesh could explain these differences.

Adolescent girls who were married or cohabiting and those who were widowed/divorced/separated had a higher prevalence ratio of adolescent pregnancy compared to those who were never in a union. These findings corroborate previous studies conducted in Uganda, Ethiopia and Nepal.^8,17,19^ This could be attributed to the early sexual debut related to early marriage and increased encounter with sexual intercourse.^20^ Studies have shown that adolescents who are in a marriage or cohabiting relationship have a lower prevalence of contraceptive use than others.^8,17,19^ In this study, about 50% of the married/cohabiting teenagers started sexual intercourse before 15 years of age. This therefore, calls for policy-level interventional actions particularly considering that law on adolescent/child marriage are still controversial in Tanzania. While marriage is prohibited below 15 years, it is permitted under religious and cultural circumstances. Interventions should also be targeted not only on adolescent girls but also elders/communities who make the marriage decision for their adolescent girls. Countries like Nepal have a law banning adolescent marriage (<20 years) since 1963, which is not the same in Tanzania.

Adolescents who started sexual intercourse before 15 years of age had a 20% higher pregnancy rate compared to those who started sexual intercourse at ≥15 years of age, which is consistent with findings from Ethiopia.^17^ In most SSA and Asian countries, early sexual debut occurs in the context of child/adolescent marriages where young girls do not have any say or choice on sexual or pregnancy issues and access to health care.^9^ Sexual practices under 15 years of age are unsafe, and most adolescents of this age do not have enough sexual and reproductive health education.^20,21^ Early sexual debut tends to lead to higher sexual risk-taking behavior, such as having multiple partners, poor contraceptive use and early pregnancy.^22,23^ Inter-sectoral and multiple comprehensive interventions are needed to address both early sexual debut and adolescent pregnancy. Key among the interventions is keeping girls in secondary schools,^16,24^ improving access to correct knowledge and skills in SRH issues, and improving access to quality AYFSRH services.

### Study Strengths and Limitations

The study utilized the national representative data, which makes the findings generalizable. This study has estimated trends and factors associated with adolescent pregnancy in Tanzania; these findings are useful and can inform policy and decision makers. Nevertheless, are several limitations that might affect our conclusions. The data on age at first sex were self-reported by adolescent girls, which might have led to recall bias and hence over-or underestimation of the effect. Furthermore, being a cross-sectional, we were not able to establish causal relationships. Additionally, our analysis was limited to looking at births and current pregnancies and did not capture adolescent pregnancies that ended in miscarriage or abortion. Context and characteristics could be different for adolescent pregnancies ending in miscarriage or abortion. However, the study has provided countrywide picture of adolescent pregnancy which could be used as a basis to conduct longitudinal in-depth investigations.

## CONCLUSION

One in four adolescent girls aged 15-19 years in Tanzania has already become pregnant. The trend of adolescent pregnancy declined from 2004 to 2010 but increased in 2016. Adolescents who were married or living together with a partner and those who started sexual intercourse before 15 years old have higher pregnancy prevalence rate, while adolescents with secondary education have lower rate of adolescent pregnancy. Intervention programs should target mainly adolescents with no formal education, prevent early marriage, discourage early sexual initiation among adolescents so as to reduce adolescent pregnancy and related complications. Qualitative studies are crucial to explore reasons for the rising trends of adolescent pregnancies, particularly between 2010 and 2015/16 THDS rounds. Furthermore, a study on adolescent pregnancy that includes miscarriage and abortion is needed.
